# Physical and Psychological Effects of Nasogastric Tube (NGT) Use in Adolescents with Anorexia Nervosa: An Exploratory Study

**DOI:** 10.3390/nu18020266

**Published:** 2026-01-14

**Authors:** Federico Amianto, Tomaso Oliaro, Francesca Righettoni, Chiara Davico, Daniele Marcotulli, Andrea Martinuzzi

**Affiliations:** 1 Neurosciences Department, University of Torino, Via Cherasco 15, 10126 Turin, Italy; 2 Department of Public Health and Pediatric Sciences, University of Turin, 10126 Turin, Italyandrea.martinuzzi@unito.it (A.M.)

**Keywords:** anorexia nervosa, nasogastric tube feeding, adolescents, tolerability, psychometrics, therapeutic alliance

## Abstract

**Background:** Anorexia nervosa (AN) may require nasogastric tube (NGT) feeding when oral intake is insufficient. Evidence on the psychological impact and prognostic correlates of NGT use in adolescents affected with AN is limited. **Methods:** Fifty-seven adolescent inpatients (96.5% female; age range 12–18 years; and mean age 15.0 ± 1.51 years) affected with AN admitted in a child psychiatry ward and treated with NGT re-feeding in addition to oral nutrition were included in the study. A 21-item VAS questionnaire was administered at intake (T0), after NGT introduction (T1), after one week of NGT use (T2), and after NGT dismissal (T3) to assess the physical and psychological effects. Participants were also assessed with psychometric measures including personality (TCI), eating psychopathology (EDI-2), general psychopathology (BDI-II, SCL-90-R, and TAS), and family functioning (FAD). The measures were compared between each timepoint with paired *t*-tests and ANOVA for repeated measures. Pearson correlations were performed between the VAS scores and psychometric measures. **Results:** From admission to discharge, weight increased by +3.2 kg and BMI by +1.2 kg/m^2^. Items 1, 3, 4, 6, 15, 18, and 20 of the VAS questionnaire items showed significant improvement over time. TCI personality traits, EDI-2 eating and BDI, SCL-90 and TAS general psychopathology, and FAD family functioning were related to NGT perception by the AN adolescents. **Conclusions:** NGT was helpful in weight progression during inpatient treatment. It was generally well tolerated, with progressive improvement in psychological and physical discomfort during treatment. The meaningful associations with specific psychometric features suggest the possibility to tailor the NGT use based on adolescent characteristics. Multidisciplinary care and tailored psychoeducation may enhance NGT acceptance.

## 1. Introduction

Anorexia nervosa (AN) is defined in the Diagnostic and Statistical Manual of Mental Disorders, Fifth Edition (DSM-5) [1], as a psychiatric illness characterized by excessive caloric restriction, an intense fear of gaining weight, and a distorted body image. The estimated worldwide lifetime prevalence rate is 0.2%, and 0.9% in Western populations, with a peak incidence in females aged 15 to 19 years [2]. However, most cases of eating disorders go undetected by the healthcare system, limiting the epidemiological understanding of this condition [3]. AN etiology is multifactorial, involving psychological, physiological, and sociocultural factors, in a complex interaction between genetics and environment [4]. AN significantly affects cognitive, emotional, and social functioning and leads to severe medical complications across multiple systems, including cardiovascular, gastrointestinal, endocrine, and cerebral [5]. Without adequate treatment, AN can be fatal. The standardized mortality ratio is 5.9 (95% CI 4.2–8.3), representing an approximately six-times-higher risk than that of the general population [6].

AN treatment requires a multidisciplinary approach (physicians, dietitians, psychologists, etc.) and the development of coordinated care plans [7]. The first step in the therapeutic path is weight restoration, which is essential for medical stabilization and the reversal of long-term complications [8]. The literature recommends oral nutrition as the first-line treatment, with the introduction of nasogastric tube feeding if dietary goals are not achieved [9]. The Medical Emergencies in Eating Disorders (MEED) guidelines support the use of NG tube feeding only as a second-line option, specifying, as criteria, clinical/biochemical instability and life-threatening weight loss (Body Mass Index or BMI < 13) [10]. Studies show that enteral nutrition via nasogastric tube ensures the rapid restoration of nutrition and reduces immediate health risks [11]. However, although recognized as an essential step for medical stabilization and the reversal of long-term complications, NGT remains a controversial procedure [12]. Its invasive nature may exacerbate body image psychopathology and potentially undermine the therapeutic alliance between patient and psychiatrist [13]. Enteral feeding may also intersect with personality traits commonly observed in the AN patient, such as perfectionism, rigidity, and a high need for control [14]. These traits can influence how patients perceive and respond to treatment, which may be experienced as invasive or coercive [15]. In the event of rapid health deterioration and severe psychopathological impairments, treatment may be carried out against the patient’s will [16]. The impact of such coercion can be highly traumatic for patients and parents, potentially leading to conflict, misunderstandings, and negative emotions [17]. NGT is often experienced as an unpleasant physical sensation and as an external control over individual freedom but also as a necessary and useful support for survival as well as a symbol of illness, forcing patients to confront the severity of their condition and prompting awareness [18].

The literature remains scarce on the psychological effects of this treatment and on the impact of enteral feeding on therapeutic alliance. Overall, the available evidence is limited: the number of patients studied is small, no validated tools have been adopted to assess the effects of NG feeding, no reports have considered medium- and long-term psychological consequences, and little attention has been paid to psychiatric comorbidity or personality traits [19]. Moreover, studies on the effects of NG tube feeding have been predominantly conducted in adults or late adolescents, while data on treatment perception in pediatric populations are extremely limited [11,12,15,16,18,19].

This study aims to evaluate the tolerability and prognostic aspects of nasogastric tube feeding in the treatment of adolescent patients with AN. Based on the data available to us and the previous literature on the subject [19], we expect to confirm the usefulness of nasogastric tube feeding in AN patients and to observe good overall tolerability. The primary objective of the study is to assess the psychological and physical impact of nasogastric tube feeding in a sample of adolescent patients with AN admitted to the Regina Margherita Children’s Hospital in Turin (OIRM). The secondary objective is to evaluate any significant correlation between the impact of nasogastric tube feeding and the clinical or psychometric characteristics of the patients.

## 2. Materials and Methods

### 2.1. Participants

This observational exploratory study is based on data collected from patients consecutively admitted to the Regina Margherita Children’s Hospital (OIRM) in Turin. Data collection took place between March 2022 and July 2024. Inclusion criteria were as follows: full AN diagnosis assessed by an expert psychiatrist through the Structured Clinical Interview for DSM-5 [20]; age > 12 and <18 years; and written informed consent/ethical approval. There were no gender restrictions. Exclusion criteria: intellectual disability or pervasive developmental disorder, any neurodevelopmental disorder, acute psychosis or/and bipolar disorder, or any other inability to understand/complete psychometric tests for clinical/language/cultural reasons.

For each participant, the following clinical data were collected: age, admission/discharge weight (kg), admission/discharge BMI, length of hospital stay (days and months), and psychiatric comorbidities. These data are presented in Table 1 (clinical and demographic data of the sample). The study adhered to the Declaration of Helsinki and was approved by the hospital Ethics Committee.

### 2.2. Intervention

A 21-item, 1–10 VAS questionnaire (Appendix A) was developed and approved by the Ethics Committee. Items 1–15 addressed psychological aspects related to NGT; items 16–21 addressed physical aspects (discomfort, distress, and pain). Patients completed the questionnaire autonomously. Administration timepoints: before insertion (T0), after insertion (T1), after the first week of treatment (T2), and after removal (T3).

### 2.3. Measures

Psychometric instruments:

#### 2.3.1. Temperament and Character Inventory (TCI) [21]

The TCI is a self-administered questionnaire with 240 true/false items that assess personality dimensions. According to Cloninger’s psycho-biological model, personality is divided into four temperamental dimensions (Novelty Seeking, Harm Avoidance, Reward Dependence, and Persistence), which are genetically determined and relatively stable over time, and three character dimensions (Self-Directedness, Cooperativeness, and Self-Transcendence), which evolve with age and maturation processes and are influenced by environmental and sociocultural factors. Cronbach’s alpha (Italian version) = 0.60–0.86.

#### 2.3.2. Eating Disorder Inventory-II (EDI-II) [22,23]

The EDI-II is a self-reported questionnaire with 91 items assessing critical traits for understanding eating disorders. It measures 11 scales: Drive for Thinness (DT), Bulimia (BU), Body Dissatisfaction (BD), Ineffectiveness (IN), Perfectionism (PF), Interpersonal Distrust (ID), Interoceptive Awareness (IA), Maturity Fears (MF), Asceticism (ASC), Impulse Regulation (IR), and Social Insecurity (SI). Cronbach’s alpha (Italian version) = 0.70–0.94.

#### 2.3.3. Beck Depression Inventory-II (BDI-II) [24]

The BDI-II is a self-report instrument that assesses depression severity in adolescents and adults with psychiatric diagnoses. It includes 21 items and provides a total score plus two subscale scores (somatic–affective and cognitive). Cronbach’s alpha (Italian version) = 0.76–0.77.

#### 2.3.4. Symptom Checklist-90-R (SCL-90-R) [25]

SCL-90-R is a self-reported questionnaire that evaluates a wide spectrum of psychological problems and psychopathological symptoms, capturing both internalizing (depression, somatization, and anxiety) and externalizing (aggression, hostility, and impulsivity) dimensions in psychiatric, general medicine, and non-clinical populations. Cronbach’s alpha (Italian version) = 0.70–0.96.

#### 2.3.5. Attachment Style Questionnaire (ASQ) [26,27]

ASQ is a self-reported questionnaire designed to measure five dimensions of adult attachment, consisting of 40 items. Cronbach’s alpha (Italian version) = 0.62–0.79.

#### 2.3.6. Toronto Alexithymia Scale (TAS-20) [28,29]

TAS-20 is a clinical questionnaire assessing alexithymia, a personality trait characterized by reduced or absent ability to differentiate, recognize, or express emotional experiences and bodily sensations. Cronbach’s alpha (Italian version) = 0.82.

#### 2.3.7. Parental Bonding Instrument (PBI) [30,31]

PBI is a self-report measure assessing the quality of past attachment relationships based on recall of parental bonding during the first 16 years of life. It consists of 25 items describing two scales, Care (12 items) and Overprotection/Control (13 items), which are considered key parental behavior dimensions. Cronbach’s alpha (Italian version) = 0.76–0.88.

#### 2.3.8. Narcissistic Personality Inventory-40 (NPI-40) [32,33]

Explores narcissism through 40 item pairs, asking subjects to choose the alternative that best describes them. Cronbach’s alpha (Italian version) = 0.81–0.83.

#### 2.3.9. Family Assessment Device (FAD) [34]

FAD is a self-report instrument consisting of 60 multiple-choice items on a 4-point Likert scale, designed to reliably assess family functioning. Cronbach’s alpha (Italian version) = 0.68–0.82.

### 2.4. Data Analysis

Mean scores (average responses of 57 participants) of clinical data, VAS questionnaire responses for each question at each timepoint, and psychometric measures were compared between timepoints using paired *t*-tests (T0–T1, T1–T2, T2–T3, and T0–T3) and ANOVA across time (T0→T3).

Pearson correlations were performed between the scores of VAS questionnaire items and clinical/psychometric measures at T0 and across their pairwise time differences (deltas).

Bonferroni correction set significance to *p* < 0.01 to reduce Type-I error. Statistical analysis was conducted using the Statistical Package for Social Sciences (SPSS 27.0).

## 3. Results

### 3.1. Characteristics of the Study Sample

The study sample consisted of 57 inpatients (55 females, 96.5%) with a DSM-5 diagnosis of AN. No patient corresponding to the recruitment criteria refused participation in the study. Table 1, Table 2 and Table 3 summarize demographic, clinical, diagnostic, and comorbidity characteristics.

### 3.2. VAS Questionnaire Evolution

Table 4 reports significant comparisons between timepoints with *p* ≤ 0.01.

Question 1, “Do you think the NGT is necessary in your situation?” showed a significant decrease between T1 and T2 (*p* < 0.009).

Question 3, “Do you feel discouraged and sad about using NGT?” showed a significant decrease between T0 and T1 (*p* < 0.002) and between T0 and T3 (*p* < 0.001), confirming a significant overall time effect with the ANOVA (*p* < 0.001).

Question 4, “Do you think the NGT is a medical imposition?” showed a significant decrease between T2 and T3 (*p* < 0.009).

Question 6, “Do you think the tube interferes with your daily activities?” showed a significant decrease between T0 and T1 (*p* < 0.002).

Question 15, “Do you feel better now compared to when you started NG feeding?” showed a significant increase between T1 and T2 (*p* < 0.003), confirming a significant overall time effect with the ANOVA (*p* < 0.001).

Question 18, “Do you experience coughing and gagging?” showed a significant decrease across T0–T3, confirming a significant overall time effect with the ANOVA (*p* < 0.006).

Question 20, “Do you feel any discomfort or sore throat due to the feeding tube?” showed a significant decrease between T1 and T2 (*p* < 0.002).

### 3.3. Clinical and Psychometric Correlations of VAS Questions at T0

Pearson correlations between questionnaire items and clinical/psychometric variables at T0 are summarized in Table 5.

The item “Do you think the NGT is necessary in your situation?” (Question 1) correlated negatively with Novelty Seeking (*p* < 0.001).

The item “Do you feel discouraged and sad about using NGT?” (Question 3) correlated negatively with Cooperativeness (*p* < 0.006) and with EDI-2 Bulimia (*p* < 0.008).

For “Do you think the NGT is a medical imposition?” (Question 4), a negative correlation was observed with EDI-2 Bulimia (*p* < 0.004).

The item “Are you comfortable using NGT?” (Question 5) correlated negatively with TCI Persistence (*p* < 0.005).

For “Do you think the NGT helps you understand the value of your nutrition?” (Question 9), a negative correlation emerged with TAS Difficulty Describing Feelings (*p* < 0.006).

The item “Do you feel regret at the idea of removing the tube?” (Question 12) showed positive correlations with EDI-2 Impulsiveness (*p* < 0.001) and with the Cognitive, Somatic, and Total BDI-II scores (*p* < 0.005).

For “Do you feel anxious about removing the NGT?” (Question 13), significant positive correlations were identified with Harm Avoidance (*p* < 0.009), EDI-2 Inadequacy (*p* < 0.008), EDI-2 Asceticism (*p* < 0.001), SCL-90 Anxiety and Total (*p* < 0.010), and with all BDI-II subscores (*p* < 0.001).

The item “Do you feel better now compared to when you started NG feeding?” (Question 15) correlated positively with Novelty Seeking (*p* < 0.001).

Finally, “Do you feel nausea/disgust due to the NGT?” (Question 21) correlated positively with the Problem Solving dimension of the FAD (*p* < 0.006).

### 3.4. Clinical and Psychometric Correlations of VAS Changes

Analyses of score variations between consecutive or cumulative time intervals revealed several significant associations with clinical and psychological measures (Table 6).

Changes in Question 1, “Do you think the NGT is necessary in your situation?” from T1 to T2 correlated negatively with the age of symptom onset (*p* < 0.008).

Variations in Question 2, “Would you like to avoid NG feeding?” from T0 to T3 showed a strong positive association with EDI-2 Bulimia (*p* < 0.003).

For Question 3, “Do you feel discouraged and sad about using NGT?” the change from T0 to T1 correlated positively with NPI-40 Vanity (*p* < 0.007).

Variations in Question 4, “Do you think the NGT is a medical imposition?” during T2–T3 correlated positively with SCL-90 Somatization, Interpersonal Sensitivity, Anxiety, Phobic Anxiety, and Total scores (*p*< 0.007), as well as with the BDI-II Somatic scale (*p* < 0.005).

Changes in Question 6, “Do you think the NGT is hindering your daily activities?” from T0 to T3 were positively associated with BMI at discharge (*p* < 0.007) and negatively associated with EDI-2 Impulsiveness (*p* < 0.009).

For Question 21, “Do you feel nausea/disgust due to the NGT?” strong positive correlations in T0–T1 were found with ASQ Concern About Social Relations (*p* < 0.001) and NPI-40 Superiority (*p* < 0.001). In the T2–T3 interval, the same item correlated positively with BMI at discharge (*p* < 0.006) and negatively with the Roles dimension of the FAD (*p* < 0.009).

## 4. Discussion

NGT is often a necessary tool in the treatment of patients with AN who refuse to eat the amount of food prescribed in the dietary plan for a prolonged period and present physical conditions that cannot tolerate further food deprivation [35]. The usefulness of NGT use has been confirmed by the present exploratory study on a representative sample of the AN population requiring inpatient treatment in the region (Piedmont, Italy), referring to the OIRM. The most evident physical effect of NGT use is the significant weight gain achieved through the combined action of enteral and natural nutrition, which confirms the literature findings [11].

Nevertheless, the aim of this study was to also investigate the physical side effects and the psychological effect of the NGT in adolescents with anorexia nervosa, since the positioning of the NGT is often considered a controversial step in the treatment of this disorder [12]. At the beginning, tube feeding can be a major source of anguish for the patients, whose greatest fear is rapid weight gain beyond their control in addition to the fear of pain during positioning and physical distress during its use. It may also represent a major source of concern and stress for parents (and even professional staff) who can be disgusted by the esthetic appearance of the tube and by the fear of discomfort for their children [13,36,37].

### 4.1. Acceptance and Psychological Adaptation to NGT

The dissent to NGT use may be related to adolescents’ and/or family and staff prejudices on its impact on their activities (e.g., studying, playing, and relating to peers). The early reduction in discouragement/sadness and of perceived NGT interference with daily activities just after positioning support this interpretation.

Moreover, some improvements are also experienced across treatment (T1–T2). Patients reported a progressive reduction in discomfort and progressive increase in wellbeing following the positioning of the NGT. This is possibly because of the positive effects of re-feeding on physical strength but also on mental functioning (including the relative de-responsabilization on weight gain and food choice) combined with the reduction in fears and discomfort related to negative prejudices. These changes possibly also reflect the process of adaptation to a medical disposition, which, at first instance, may be perceived as an imposition. This is in agreement with the literature which shows that, in general, AN patients tolerate NGT both physically and psychologically [38].

### 4.2. Perceived Medical Imposition and Therapeutic Alliance

The late (T2–T3) reduction in AN adolescent’s perception of the NGT as a medical imposition has relevant implications. The literature has described NGT as an external control that may threaten autonomy, especially when restraints have been used for insertion against a patient’s will [18,37,39]. Adolescents in our sample demonstrate that the reduction in negative prejudices and the improvement in physical and psychological wellbeing led them to understand that the NGT was a support given to them to optimize their treatment. This possibly improved the perception of the alliance with the medical staff, which is in itself a goal in the treatment of this disorder [9,40]. Moreover, along with subjective wellbeing improvement and weight restoration, adolescents reduce the perception of the necessity of the NGT. This suggests that, after the first initial negative sense of coercion, they perceive the NGT more realistically, but they do not become excessively dependent on it. In fact, despite their adaptation, patients continued to express a constant desire to avoid using the NGT (Q2), with no significant decrease during treatment. Clear, upfront communication and consistent psychological support given by the staff may mitigate coercion perceptions and strengthen the alliance from the start of NGT treatment. Emphasizing collateral benefits (e.g., reduced binge–purging in patients on tube therapy) may help transform fear into trust [11,12,15,19].

### 4.3. Physical Discomfort and Symptom Management

In addition to the clear benefits of weight gain, the physical distress caused by the NGT positioning also improves during its use. Some physical symptoms (e.g., coughing/gagging; sore throat) decreased significantly, even if they did not fully resolve. This evidence implies that the improvement of NGT perception does not derive from a complete absence of discomfort but from a balance between the perceived benefits and the unavoidable discomforts, as happens with any medical treatment. On the other hand, this underscores the importance, along with all the treatment, of optimizing volumes, rates, and caloric targets to reduce nausea, vomiting, dumping, and re-feeding risks [36].

### 4.4. Correlation of NGT Perception and Evolution with Baseline Clinical Features

The perception of the need for NGT use (Q1) varies depending on the age at which patients first developed symptoms of AN. Patients with an earlier onset appear to have greater difficulty recognizing the tube as necessary. Nevertheless, there is also an evident progression in their perception during treatment, which is greater for those with a lower age of onset. This may indicate less insight into the severity of the disease in younger patients and suggests that, in these adolescents in particular, there is a need for targeted psychoeducational interventions. This will help them understand the importance of artificial re-feeding to foster the treatment and to avoid the chronic evolution of the disorder [3,5].

The greater the BMI at is discharge, the greater the improvement of nausea and of the impact on daily activities is. On one hand, physical symptoms like nausea and the limitations of daily activities may impair the nutritional benefits of NGT. This underscores that the constant attention given to reducing negative physical symptoms related to artificial re-nutrition may be crucial to improve NGT results [36]. On the other hand, it is also possible that the gradual success of re-nutrition may help to overcome the negative NGT perception and facilitate the reactivation of adolescents’ activities.

### 4.5. Correlation of NGT Perception and Evolution with Baseline Personality Features

Personality characteristics of individuals with AN can influence how patients perceive and respond to treatments [41] including the acceptance of NGT. A higher NS, which implies a less rational approach to care [21], may impair the awareness of the need for the NGT use, but, on the other hand, it increases the perception of improvement due to its application, suggesting that greater psychoeducational efforts before its positioning may be balanced by a greater final satisfaction after its use [15,19]. HA, a trait which is strictly related to feelings of anxiety and depression [21], may increase the fears associated with the suspension of NGT treatment, making individuals with HA deserving of specific reassurance by the staff [19]. The higher the persistence is, a personality trait which impairs adaptation to changes [21], the greater the perceived discomfort associated with NGT use is, while greater cooperativeness reduces the perception of the NGT as a medical imposition, stressing the pivotal role of therapeutic alliance [9,40].

Finally, also some narcissistic personality traits are related to the changes in some negative perceptions of the NGT [32,33]. The greater the vanity is, the greater the initial improvement in the discouragement created by the NGT is, possibly because the negative perception of the NGT as impairing the physical aspect is rapidly overcome after its positioning. On the other hand, the greater the sense of superiority, the greater the reduction in the sense of nausea after NGT use.

### 4.6. Correlation of NGT Perception and Evolution with Baseline Psychopathology Features

Psychiatric co-morbidities such as anxiety disorders and mood disorders may affect subjective responses to stressors related to nasogastric tube feeding [42,43]. In our study specific psychopathology traits relate to the quality of patients’ experiences with the NGT. Patients with bulimic traits showed a less discouraged attitude at the beginning of tube treatment, perceiving, to a lesser degree, the NGT as medical coercion. The greater the bulimia is, the greater the progressive improvement of NGT refusal is. This is consistent with some literature reports suggesting that the use of the NGT may be welcomed by patients with binging–purging symptoms [11,12,15,19]. Their need for control [15] may make them perceive the NGT as a substantial help in controlling their symptoms.

According to the literature [42,43], both somatic and cognitive depression and anxious features, associated with a greater general psychopathology, sense of inadequacy, and greater asceticism, are related to a poorer tolerance of the end phase of treatment. The AN adolescents affected by these features may perceive themselves as more fragile and less prepared to face medical requests, may have more difficulty managing change, and may have a greater fear of loss [42]. On the other hand, the greater the initial psychopathology, the greater the improvement in the negative perception of the NGT as a medical imposition is, suggesting that psychopathology may have a role in hindering the therapeutic alliance. This suggests the need to identify patients with traits of anxiety and depression to provide them with appropriate treatment (possibly also pharmacological) and constant emotional and psychological support until the end of treatment [40,44], in addition to transparent communication and involvement in decision-making processes [9,19].

### 4.7. Correlation of NGT Perception and Evolution with Baseline Family Features

The relationship between the perception of nausea and family difficulties in problem solving and the definitions of roles emphasizes that the psychological effect of the NGT on patients with AN cannot be understood solely through a medical approach. Rather, it is also influenced by individual psychological characteristics and family dynamics. Especially in AN, the illness of one member involves the entire family [45]. The family context can amplify or mitigate the NGT’s negative perception. A reduced ability of the family in problem solving, eventually coupled with overprotective or dysfunctional attitudes, may make the acceptance of the NGT more complex, with greater risks of negative somatic symptoms [19]. The literature suggests that, particularly for adolescents with AN, a treatment approach involving the patient’s entire family is more effective than other psychotherapeutic treatments based on physical recovery markers, satisfaction with services, and therapies received [44]. A multidisciplinary approach that integrates medical treatment with psychological and family support is needed to improve both the acceptance and effectiveness of the NGT in patients with AN [45,46,47,48].

### 4.8. Study Limitations and Future Directions

The is an exploratory, observational, single-arm study based on a sample coming from a single center and includes a relatively small sample of inpatients, with a clear predominance of girls. The lack of a control group (e.g., patients treated exclusively orally) makes it impossible to determine whether the observed changes are specific to the use of NGT or reflect the general effects of hospitalization, re-feeding, and intensive therapeutic care. Moreover, it includes very malnourished patients at risk of refeeding syndrome but does not differentially explore the treatment effect in those patients.

The 21-item VAS questionnaire, although developed for the study and approved by the bioethics committee, has no psychometric validation; thus, the interpretation of individual items in the questionnaire and their changes over time remains descriptive and hypothetical.

We applied numerous statistical tests and an extensive correlation analysis with many psychometric variables: despite the use of Bonferroni correction, a high risk of Type-I error cannot be excluded. Moreover, there are no multivariate models to allow for a more controlled interpretation of the relationships; thus, many correlations may be random or indirect and should not be interpreted as causal.

Limitations of this study also include potential test–retest learning effects and Hawthorne effects. Larger, controlled, longitudinal studies with validated instruments specific to NGT perceptions and long-term follow-up are needed to confirm and give the correct interpretation to our results.

## 5. Conclusions

NGT in adolescent AN was associated with meaningful weight/BMI restoration and a general trajectory of improved psychological and physical tolerability across treatment. Significant correlations were also found between improvements in BMI and quality of life and therapeutic alliance at hospital discharge [49]. Perceptions of necessity, coercion, and discomfort were modulated by personality, symptom burden, and family functioning. Tailored psychoeducation, alliance-focused communication, and family involvement may optimize acceptance and clinical outcomes. Researchers have focused on evaluating the motivation to change in relation to therapeutic alliance.

## Figures and Tables

**Table 1:** Demographic characteristics of the sample.

Variable	N (%)
Sample size (*n*)	57
Gender—Female	55 (96.5%)
Gender—Male	2 (3.5%)
	M ± SD
Mean age at admission (years)	15.0 ± 1.51
Symptoms onset (years)	13.1 ± 1.57

**Table 2:** Clinical characteristics of the sample.

Variable	Admission (T0) M ± SD	SNG Removal (T3) M ± SD	ΔT0–T3 M ± SD	*p*
Weight (kg)	37.9 ± 7.04	41.1 ± 5.85	+3.2 ± 2.46	0.000
BMI (kg/m^2^)	14.7 ± 2.33	15.9 ± 1.58	+1.2 ± 1.09	0.000
Length of stay (days)	—	61.1 ± 26.25	—	—

**Table 3:** Primary diagnosis at symptoms onset and psychiatric comorbidity.

Diagnosis	N (%)
Anorexia Nervosa—restricting type	42 (73.7)
Anorexia Nervosa—binge–purging type	3 (5.2)
Anorexia Nervosa—purging type	12 (21.0)
**Comorbid Diagnosis**	
Major Depressive Disorder	34 (59.6)
Generalized Anxiety Disorder	18 (31.6)
Obsessive–Compulsive Disorder	17 (29.8)
Self-harming/Parasuicidal Behaviors	13 (22.8)
Panic Disorder	11 (19.3)
Specific Learning Disorder	3 (5.2)
Bipolar Disorder	4 (7.0)
Schizophrenia	2 (3.5)

**Table 4:** *T*-test and ANOVA comparison between timepoints.

***T*-TEST ANALYSIS**						
**Question**	**Period**	**N**	**M1 ± SD**	**M2 ± SD**	**t**	***p***
1. Do you think NGT is necessary in your situation?	T1–T2	30	6.21 ± 3.38	4.94 ± 3.18	2.798	0.009
3. Do you feel discouraged and sad about using NGT?	T0–T1	25	7.33 ± 2.66	5.91 ± 3.26	3.395	0.002
	T0–T3	18	7.33 ± 2.66	4.50 ± 3.03	3.937	0.001
4. Do you think the NGT is a medical imposition?	T2–T3	18	6.73 ± 3.16	5.61 ± 3.33	2.786	0.009
6. Do you think the tube interferes with your daily activities?	T0–T1	25	5.33 ± 2.83	4.27 ± 3.00	3.547	0.002
15. Do you feel better now compared to when you started NG feeding?	T1–T2	30	4.47 ± 2.84	6.00 ± 2.84	−3.167	0.003
20. Do you feel any discomfort or sore throat due to the feeding tube?	T1–T2	30	6.27 ± 3.14	5.15 ± 3.30	3.462	0.002
**ANOVA ANALYSIS**						
**Question**	**Period**	**N**	**M1 ± SD**	**M2 ± SD**	**F**	***p***
3. Do you feel discouraged and sad about using NGT?	T0–T3	18	7.33 ± 2.66	4.50 ± 3.03	6.939	0.001
15. Do you feel better now compared to when you started NG feeding?	T0–T3	18	4.17 ± 2.40	6.33 ± 2.81	6.031	0.001
18. Do you experience coughing and gagging?	T0–T3	18	3.33 ± 2.70	1.72 ± 1.56	4.714	0.006

Note: M1 = mean of the first timepoint; M2 = mean of the second timepoint. Timepoints: T0 = before insertion; T1 = after NGT insertion; T2 = after a week of treatment; and T3 = after removal.

**Table 5:** Correlations between NGT-related questions and psychometric characteristics (T0).

	Psychometric Features	r	*p*
1. Do you think NGT is necessary in your situation?	Novelty Seeking (TCI)	−0.614	0.001
3. Do you feel discouraged and sad about using NGT?	Cooperativeness (TCI)	−0.543	0.006
	Bulimia (EDI-2)	−0.525	0.008
4. Do you think the NGT is a medical imposition?	Bulimia (EDI-2)	−0.565	0.004
5. Are you comfortable using NGT?	Persistence (TCI)	−0.549	0.005
9. Do you think the NGT helps you understand the value of your nutrition?	Difficulty Describing Feelings (TAS)	−0.543	0.006
12. Do you feel regret at the idea of removing the tube?	Impulsiveness (EDI-2)	0.641	0.001
	Cognitive Depression (BDI-II)	0.559	0.005
	Somatic Depression (BDI-II)	0.593	0.002
	Total (BDI-II)	0.579	0.003
13. Do you feel anxious about removing the NGT?	Harm Avoidance (TCI)	0.521	0.009
	Inadequacy (EDI-2)	0.526	0.008
	Asceticism (EDI-2)	0.622	0.001
	Anxiety (SCL-90)	0.612	0.001
	SCL-90 Total	0.513	0.010
	Cognitive Depression (BDI-II)	0.644	0.001
	Somatic Depression (BDI-II)	0.689	<0.001
	Total (BDI-II)	0.664	<0.001
15. Do you feel better now compared to when you started NG feeding?	Novelty Seeking (TCI)	0.661	<0.001
21. Do you feel nausea/disgust due to the NGT?	Problem Solving (FAD)	0.547	0.006

Note: TCI = Temperament and Character Inventory; EDI-2 = Eating Disorder Inventory-2; BDI-II = Beck Depression Inventory-II; FAD = Family Assessment Device; SCL-90 = Symptom Checklist-90.

**Table 6:** Significant correlations between question changes and psychometrics.

Question	Δ Period	N	Characteristic	r	*p*
1. Do you think NGT is necessary in your situation?	T1–T2	33	Age of symptom onset	−0.456	0.008
2. Would you like to avoid NG feeding?	T0–T3	11	Bulimia (EDI-2)	0.805	0.003
3. Do you feel discouraged and sad about using NGT?	T0–T1	16	Vanity (NPI-40)	0.649	0.007
4. Do you think the NGT is a medical imposition?	T2–T3	21	Somatizations (SCL-90)	0.572	0.007
			Interpersonal sensitivity (SCL-90)	0.602	0.004
			Anxiety (SCL-90)	0.627	0.002
			Phobic anxiety (SCL-90)	0.618	0.003
			Total (SCL-90)	0.602	0.004
			Somatic depression (BDI-II)	0.589	0.005
6. Do you think the NGT is hindering your daily activities?	T0–T3	18	BMI at discharge	0.608	0.007
			Impulsiveness (EDI-2)	−0.742	0.009
21. Do you feel nausea/disgust due to the NGT?	T0–T1	16	Preoccupation with relationships (ASQ)	0.790	<0.001
			Superiority (NPI-40)	0.732	0.001
	T2–T3	30	BMI at discharge	0.491	0.006
		20	Roles (FAD)	−0.567	0.009

Note: NPI-40 = Narcissistic Personality Inventory-40; EDI-2 = Eating Disorder Inventory-2; BDI-II = Beck Depression Inventory-II; FAD = Family Assessment Device; SCL-90 = Symptom Checklist-90; ASQ = Attachment Style Questionnaire.

## Data Availability

The data presented in this study are available on request from the corresponding author due to privacy reasons.

## Abbreviations

The following abbreviations are used in this manuscript:

TCI	Temperament and Character Inventory
NPI-40	Narcissistic Personality Inventory-40
EDI-2	Eating Disorder Inventory-2
BDI-II	Beck Depression Inventory-II
FAD	Family Assessment Device
SCL-90	Symptom Checklist-90
ASQ	Attachment Style Questionnaire
TAS-20	Toronto Alexithymia Scale 20
NS	Novelty Seeking
HA	Harm Avoidance
P	Persistence
C	Cooperativeness

## Appendix A

QUESTIONNAIRE ON NASOGASTRIC TUBE (NGT) USE

Name and surname: ______________________________

Date: ______________________________

Week of treatment: ______________________________

**Instructions:**

This questionnaire aims to investigate how you experience treatment with a nasogastric tube (NGT), both physically and psychologically.

For each question, select a number from 0 to 10 that best represents your experience (0 = not at all; 10 = extremely).

**Example (do not answer): Do you feel tired?**

Please circle one number:

0	1	2	3	4	5	6	7	8	9	10
□	□	□	□	□	□	□	□	□	□	□

#	Question	Score (0–10)										
1	Do you think the nasogastric tube (NGT) is necessary in your situation?	0	1	2	3	4	5	6	7	8	9	10
2	Would you like to avoid using the NGT?	0	1	2	3	4	5	6	7	8	9	10
3	Do you feel discouraged and sad because you have to use the NGT?	0	1	2	3	4	5	6	7	8	9	10
4	Do you think the NGT is a medical imposition?	0	1	2	3	4	5	6	7	8	9	10
5	Do you feel comfortable with the NGT?	0	1	2	3	4	5	6	7	8	9	10
6	Do you think the NGT interferes with your daily activities?	0	1	2	3	4	5	6	7	8	9	10
7	Do you think nutrition with food or oral supplements would be better?	0	1	2	3	4	5	6	7	8	9	10
8	Does the proposal to use the NGT make you feel more understood?	0	1	2	3	4	5	6	7	8	9	10
9	Do you think the NGT helps you understand the importance of your nutrition?	0	1	2	3	4	5	6	7	8	9	10
10	Does using the NGT reduce feelings of guilt about your eating?	0	1	2	3	4	5	6	7	8	9	10
11	Does using the NGT make you feel more protected?	0	1	2	3	4	5	6	7	8	9	10
12	Do you feel upset at the idea of removing the NGT?	0	1	2	3	4	5	6	7	8	9	10
13	Do you feel anxious at the idea of removing the NGT?	0	1	2	3	4	5	6	7	8	9	10
14	Has the quality of your sleep changed since the NGT was inserted?	0	1	2	3	4	5	6	7	8	9	10
15	Do you feel better now compared with when you started NGT treatment?	0	1	2	3	4	5	6	7	8	9	10
16	Do you feel pain in your nose because of the NGT?	0	1	2	3	4	5	6	7	8	9	10
17	Do you have trouble breathing through your nose because of the NGT?	0	1	2	3	4	5	6	7	8	9	10
18	Do you experience coughing and episodes of vomiting?	0	1	2	3	4	5	6	7	8	9	10
19	Do you experience the following symptoms: abdominal cramps, abdominal bloating, and diarrhea?	0	1	2	3	4	5	6	7	8	9	10
20	Do you feel discomfort or pain in your throat because of the NGT?	0	1	2	3	4	5	6	7	8	9	10
21	Do you feel nausea/disgust because of the NGT?	0	1	2	3	4	5	6	7	8	9	10

*The following section should be completed by medical staff and concerns nutritional changes in the patient’s NGT feeding.*

22. How is the patient’s current nutritional intake structured?

______________________________________________________________________________

______________________________________________________________________________

______________________________________________________________________________

______________________________________________________________________________

23. Has the content of the NGT feeding changed compared with the previous week? If yes, how?

______________________________________________________________________________

______________________________________________________________________________

______________________________________________________________________________

______________________________________________________________________________

______________________________________________________________________________
